# Cost of Mating and Insemination Capacity of a Genetically Modified Mosquito *Aedes aegypti* OX513A Compared to Its Wild Type Counterpart

**DOI:** 10.1371/journal.pone.0026086

**Published:** 2011-10-11

**Authors:** Irka Bargielowski, Luke Alphey, Jacob C. Koella

**Affiliations:** 1 Faculty of Natural Sciences, Imperial College London, London, United Kingdom; 2 Oxitec Limited, Oxford, United Kingdom; 3 Department of Zoology, University of Oxford, Oxford, United Kingdom; Institut national de la santé et de la recherche médicale - Institut Cochin, France

## Abstract

The idea of implementing genetics-based insect control strategies modelled on the traditional SIT is becoming increasingly popular. In this paper we compare a genetically modified line of *Aedes aegypti* carrying a tetracycline repressible, lethal positive feedback system (OX513A) with its wild type counterpart with respect to their insemination capacities and the cost of courtship and mating. Genetically modified males inseminated just over half as many females as the wild type males during their lifetime. Providing days of rest from mating had no significant effect on the total number of females inseminated by males of either line, but it did increase their longevity. Producing sperm had a low cost in terms of energy investment; the cost of transferring this sperm to a receptive female was much higher. Continued mating attempts with refractory females suggest that males could not identify refractory females before investing substantial energy in courtship. Although over a lifetime OX513A males inseminated fewer females, the number of females inseminated over the first three days, was similar between males of the two lines, suggesting that the identified cost of RIDL may have little impact on the outcome of SIT-based control programmes with frequent releases of the genetically modified males.

## Introduction

Strategies based on genetic manipulation are becoming more popular in the search for effective techniques for vector control. Advances in the technology for genetic transformation have made such methods feasible for the control of *Aedes aegypti*, the most important vector of dengue fever, yellow fever and other arboviruses [1]. One possible approach is the release of insects carrying a dominant lethal (RIDL) [2], [3], [4], [5], a control strategy modelled on the traditional sterile insect technique (SIT). That this strategy is feasible, in the sense that strains with the necessary novel genetic properties can be constructed, has been demonstrated with lines of *Ae. aegypti* transformed with a dominant lethal gene that can be repressed with tetracycline [6], [7]. These lines can be reared in the laboratory by adding tetracycline to the larval diet [8]; however, in nature tetracycline is not readily available, so the lethal system is activated. Transformed males, homozygous for the lethal construct, would then pass one copy of the dominant lethal gene to their offspring by normal Mendelian inheritance and these would consequently die as larvae or pupae. Mathematical models indicate that the continued releases of such ‘sterile’ males would in time lead to the suppression, or elimination, of the targeted mosquito population [9], [10]. As releasing large numbers of females would increase biting nuisance and the transmission of disease, deliberate releases of mosquitoes, even sterile ones, should be restricted to males.

The success of such a control programme is influenced by the likelihood that the released males can inseminate females and father offspring. This in turn depends on two factors – the outcome of competition between wild type and RIDL males for access to females and the relative longevities of wild type and RIDL males.

Female *Aedes aegypti* are monogamous [11], [12], i.e. mate only once and are generally refractory to a second insemination. Males, in contrast, are polygamous (e.g. [13], [14], [15], [16]) and can inseminate several females over the course of their lifetimes. The number of females is limited, with estimates of, on average, 3 to 5.8 [13], [16] females in a day and 8 to 9 over the course of a lifetime [15]. That males can inseminate more females in their lifetimes than they can in a single day suggests that their sperm reserves (or indeed reserves of other seminal fluid components) are depleted by successive matings and must be replenished before they can inseminate further females. The production of sperm, the replenishment of sperm reserves and the effort involved in courting or competing for access to females undoubtedly require energy and are therefore costly to males [17], [18], [19], [20], [21], [22]. Investment in activities relating to mating success may therefore trade off against other fitness-determining traits, such as longevity. This has been demonstrated in other species, including *Drosophila* [17] and the mosquito *Sabethes cyaneus* [22].

In this paper we compare the insemination capacity of males (i.e. the number of females a male is capable of inseminating over the course of his lifetime) and the cost of investing in courtship and mating on longevity for two mosquito colonies: a wild type strain of Malaysian origin (‘WT’) and an engineered version of this strain which is homozygous at a single locus for repressible-lethal construct (‘OX513A’).

## Methods

### Mosquito lines

#### Wild type line (WT)

The WT line originates from field-caught *Aedes aegypti* from Jinjang, Selangor, Malaysia. It was colonised in 1975. It is therefore likely to be highly lab-adapted and correspondingly perhaps poorly representative of field bred males; however it was chosen because of its genetic similarity to the modified OX513A line (see below).

#### RIDL line (OX513A)

OX513A is a homozygous RIDL line of *Ae. aegypti*, transformed with a tetracycline repressible, lethal positive feedback system [6]. A tetracycline-repressible transcriptional transactivator (tTAV) [23], [24] under the control of its own binding site (tetO) creates a positive feedback loop. The addition of tetracycline leads tTAV to bind tetracycline, in which form tTAV can no longer bind to tetO and the cycle is interrupted [6].

Mosquitoes of this line are identifiable by red fluorescence due to the expression of DsRed2 under the control of an Act5C promoter [6].

The OX513A line was repeatedly out-crossed to the WT line, so that 97%–99% of the genomes of the two experimental lines should be shared; expected exceptions are sequences closely linked to the transgene insertion and, of course, the transgene itself.

### Larval rearing

All experiments were conducted in a temperature-controlled insectary at 27 (+/−2) °C and a relative humidity of 65 (+/−10) % with a 12 h∶12 h light/dark cycle.

Eggs of the WT line and the genetically altered OX513A line were submerged in water and placed under low pressure for one hour to ensure synchronous hatching. The following day the larvae were placed in individual wells of 12-well plates with 3 ml of water per well and reared on the following regime of finely crushed TetraMin fish food; day 1: 0.06 mg, day 2: 0.08 mg, day 3: 0.16 mg, day 4: 0.32 mg, day 5: 0.64 mg, day 6 and thereafter: 0.32 mg/larva. Fish food was prepared in a tap water solution, mixed to a uniform suspension with a magnetic stirrer and aliquoted into the wells (150 µl per well) daily. The water in wells containing OX513A larvae was supplemented with 30 µg/ml tetracycline. This rearing method was chosen to produce adults (in particular males) of equivalent size for the two strains, while enabling a large number of independent repeats to be reared within a relatively small space and limited time period.

### Insemination capacity/longevity

One hundred mating arenas were set up, each a cage of 15×15×15 cm. One day after emergence, a WT or an OX513A male was placed into a cage. The fifty cages of each strain were treated in two ways. (i) Five virgin WT females were placed in the cage for ninety minutes every day until the male had died. The females were then removed, dissected and their spermathecae assayed for the presence of sperm. (ii) The second treatment differed from the previous one in that on the fourth and fifth day within consecutive 5-day periods, no females were placed into the cage, so that the males had 2 days of ‘rest’.

As only a limited number of dissections could be carried out in a day, the experiment was divided into five consecutive blocks of twenty cages, ten containing wild type males and ten containing OX513A males, half of each with rest days and half without.

### Cost of mating/longevity

To compare the effect on male longevity of increasing the number of available females the following cages were set up: 220 cages, half containing one WT male and half one OX513A male. Fifty males of each line were kept in isolation, without the addition of females. Thirty males of either line were presented either two or four virgin WT females daily. Dead mosquitoes were removed from their pots and stored for wing length measurements.

As males may be more active when they encounter virgins than previously mated (and therefore refractory) females, we compared the effect of courting receptive (virgin) and refractory (previously inseminated) females on the longevity of WT and OX513A males by setting up an additional 120 cages. Again, 30 males of either line were held with two or four refractory females. We selected WT females that had been observed to copulate with other males. In six cages, some of the females died before the male, and were replaced with females from cages of our standard colony that had not yet blood fed, but which had probably mated.

The adult mosquitoes were supplied with a piece of cotton wool saturated with a 10% sucrose solution, which was refreshed every other day to prevent desiccation. Mosquitoes were checked daily for survival.

In order to supply enough virgin females to make the daily replacements, three large trays (1 litre) of WT larvae were reared in succession at low density (approx. 0.3 larva/ml) on the same food regime as above. The pupae were sexed and transferred to female stock cages.

### Wing length measurement

Mosquitoes used for wing length measurements were put into 1.5 ml Eppendorf tubes and frozen. The wings were removed in a 70% ethanol solution under a dissection microscope and mounted on microscope slides. Digital images of the wings were taken with a Canon PowerShot S5IS camera and a 99 mm adapter (S/N:3754, Martin Microscope Company). Wings were measured with ImageJ (http://rsbweb.nih.gov/ij/) from the auxiliary incision to the apical margin excluding the fringe.

### Statistical analysis

Statistical analyses were performed with JMP version 7.0 (http://www.jmpdiscovery.com).

The number of females inseminated and longevity were analysed with an ANOVA including line (WT, OX513A), treatment (with or without rest days) and their 2-way interactions as factors. The longevity of males caged with increasing numbers of females was analysed with an ANOVA including line (WT, OX513A), number of females (0, 2, and 4) and their 2-way interactions as factors. The difference in longevity between males of either line caged with virgin or refractory females was analysed as a three-way ANOVA including (line (WT, OX513A), number of females (2, 4), kind of female (virgin, refractory) and their interactions as factors. Each analysis included block as a nominal factor. The residuals of all analyses were close to Gaussian distributed, justifying the use of the ANOVAs.

## Results

**Figure 1:**
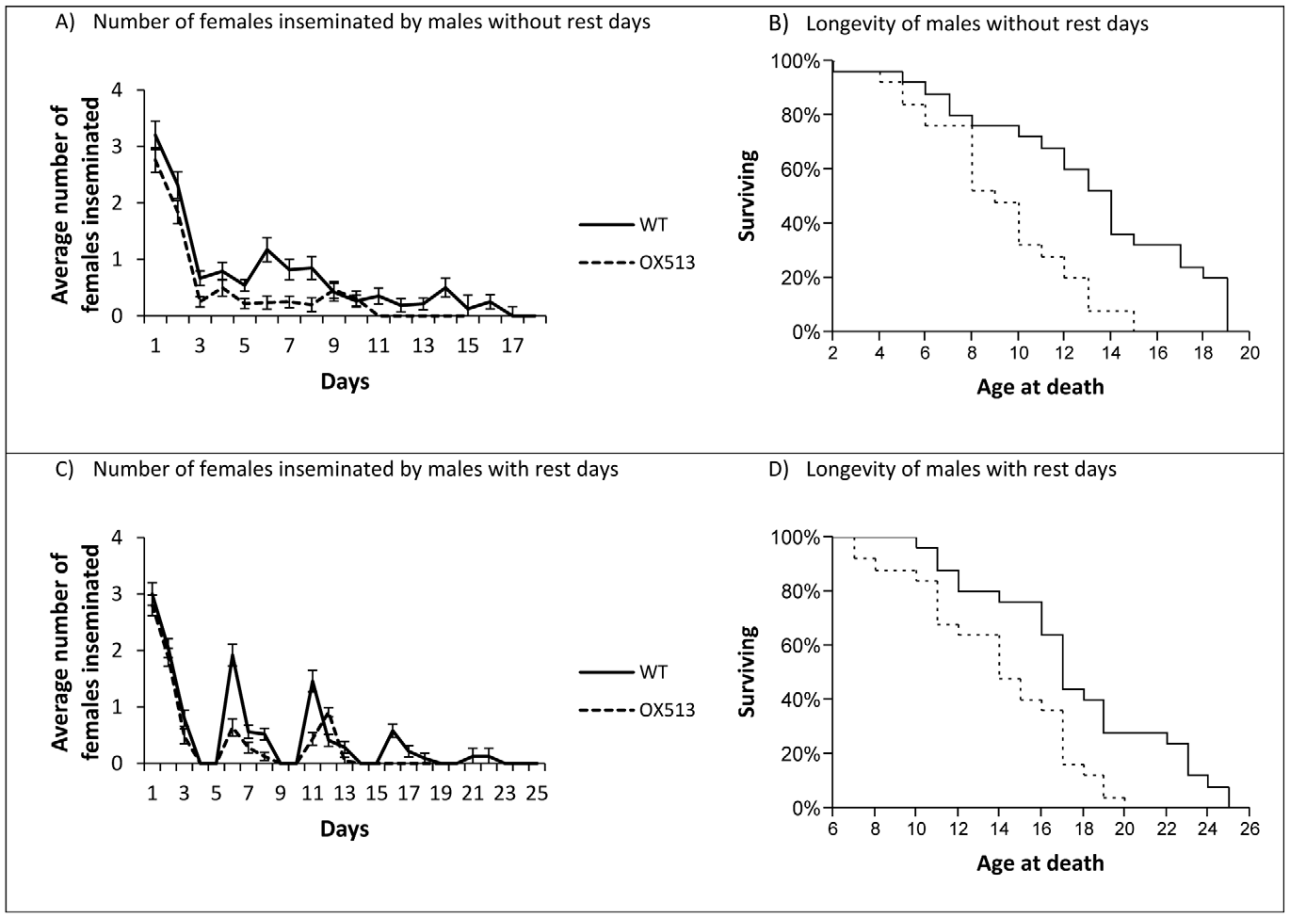
Number of females inseminated by, and longevity of males with and without rest days. WT males (solid line) inseminate more females than OX513A males (dashed line) both without (panel A)) and with (panel C)) rest days. Data points were calculated with the number of males that survived each day. WT males outlive their OX513A counterparts whether given rest days (panel D)) or not (panel B)). Error bars represent the standard error.

The longer a male of each line lived the more females he inseminated over the course of his lifetime (F = 30.4, df = 1, p<0.001). There was no difference among blocks (F = 1.3, df = 1, p = 0.263). WT males inseminated more (11.5±0.53 SE) females than OX513A males (6.6±0.31 SE) (F = 61.6, df = 1, p<0.001). Rest days, i.e. days on which no females were introduced, had no effect on the total number of females inseminated by males of each line (F = 0.05, df = 1, p = 0.823), and the difference between the two lines was not affected by the availability of rest days (interaction: F = 0.03, df = 1, p = 0.873). Blocking had no effect (F = 0.0005, df = 1, p = 0.982). WT males, regardless of whether rest days were offered, outlived OX513A males by approximately four days (F = 19.8, df = 1, p<0.001); introducing ‘rest days’ increased the average lifespan for both lines by approximately four days (F = 32.8, df = 1, p<0.001) (Figure 1), and the difference between the lines was not affected by the availability of rest days (F = 0.005, df = 1, p = 0.943). Again, blocking showed no significant effect (F = 1.269, df = 1, p = 0.263).

### Longevity of males

#### Number of females

Providing males (WT and OX513A) with virgin females reduced their average lifespan by 43% from 34.35 (±0.84 SE) to 14.62 (±0.74 SE) days (F = 313.6, df = 1, p<0.001).

**Figure 2:**
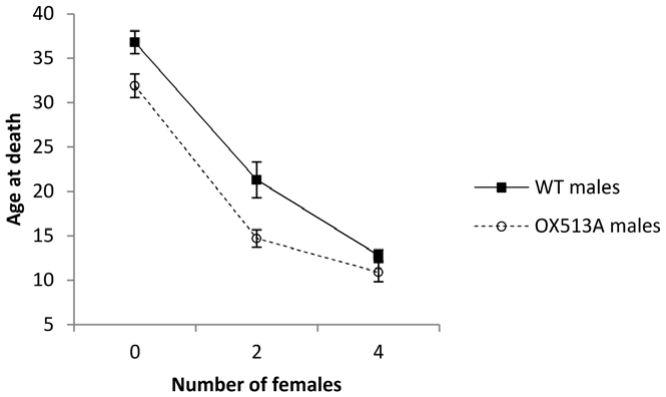
Average longevity of WT and OX513A males caged with virgin females. The longevity of WT (solid line, solid squares) and OX513A (dashed line, open circles) males decreased similarly with increasing numbers of virgin females (provided daily). Error bars represent the standard error.

The longevity of males decreased with the number of virgin females added from 34.35 (±0.95 SE) days with 0 females to 11.99 (±0.59 SE) days with 4 females (Figure 2) (F = 17.0, df = 2, p<0.001). The number of females affected males of the two lines similarly (F = 1.45, df = 2, p = 0.237) (Figure 2).

#### Mating status of females

**Figure 3:**
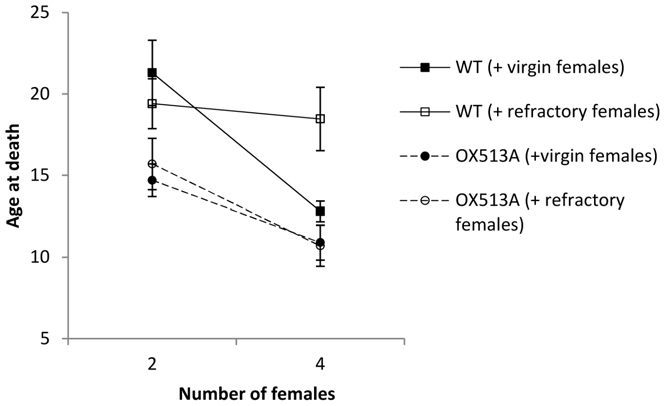
Effect of the three-way interaction on the longevity of males. Adding virgin (closed symbols) or refractory females (open symbols) has a similar effect on OX514A males (dashed lines, circles), but only adding virgin females substantially reduced the longevity of WT males (solid lines, squares). Error bars represent the standard error.

As above, the male's longevity was higher if he was caged with 2 rather than with 4 females (F = 20.95, df = 1, p<0.001), and higher for WT than for OX513A males (F = 25.12, df = 1, p<0.001). Whether the females he was caged with were virgin or refractory had only a slight effect (F = 1.314, df = 1, p = 0.253), but the interaction of these factors was significant (F = 4.843, df = 1, p = 0.029): adding virgin or refractory females has a similar effect on OX513A males, but only adding virgin females substantially reduced the longevity of WT males (Figure 3). None of the other two-way interactions were significant.

### Effect of body size on longevity

There was no difference between the average wing lengths of WT males (2.096±0.012 mm) and OX513 males (2.096±0.12 mm) (df = 1, F = 0.0001, p = 0.992) or between the average wing lengths of WT females (2.63±0.011 mm) and OX513 females (2.62±0.011 mm) (df = 1, F = 1.2097, p = 0.274). Within the limited range of body sizes produced, there was no effect on longevity.

## Discussion

This paper compares the insemination capacity of males of a genetically modified and a related wild type line of *Aedes aegypti*, as well as assessing the contributions of mating attempts and successful insemination to the overall cost of mating. Female *Ae. aegypti* are considered to be monogamous [11], [12], i.e. mate only once in their lifetime, after which they become refractory to further insemination. Keeping males with previously inseminated females will therefore principally measure the cost of futile attempts at courtship and mating. Conversely, presenting males with virgin females daily will give an indication of the cost of both coupling and successful insemination, therefore allowing us to assess the contribution of both factors to the overall cost of mating. Our experimental design does not include male contest competition although this may play a role in a more natural setting (reviewed in [25]), nor the effort required to find females dispersed in a large area. Our estimates of the costs of mating and reproduction to male *Ae. aegypti* are therefore conservative and may well increase in a field situation.

We have examined an engineered line intended for use in a population suppression strategy [4], [6], [26]. However, the same issues of fitness arise with other proposed uses of modified mosquitoes, such as attempts to make wild populations less able to transmit specific pathogens (‘refractory insects’, [27], [28]). Indeed, since such insects are intended to establish and persist in the wild, whereas sterile males are merely expected to mate and die, male mating ability may be just one of a much wider range of relevant fitness traits affecting the performance and effectiveness of refractory insects.

Our results show distinct differences in the insemination capacity and the cost of mating in males of the genetically modified OX513A and the WT line. Genetically modified males inseminated just over half as many females (on average 6.6) as the WT males (on average 11.5) during their lifetime. Providing days of rest from mating had no significant effect on the total number of females inseminated by males of each line, yet it did increase their longevity. In line with previous studies [29], keeping males confined with females significantly reduced their lifespan, and increasing the number of females a male was caged with further decreased his longevity. OX513A males, caged with refractory females, showed a greater reduction in longevity with increasing numbers of females than the WT males. Attempting to mate therefore appears more costly in terms of energy investment to the genetically modified males. On the other hand, it was the longevity of WT males that decreased to a greater extent when males were kept with increasing numbers of virgin females. This may seem counterintuitive, but as stated above the WT males are capable of inseminating almost double the number of females than the genetically modified males. Furthermore, Jones [30] noted that sperm depleted males no longer attempted mating to the extent of ‘fresh’ males. It is therefore conceivable that the OX513A males which ran out of sperm more quickly reduced their mating efforts sooner than the WT males.

The reduction in longevity differed between WT and OX513A males housed with previously inseminated females and males presented with virgin females daily. While OX513A males showed no difference in longevity when housed with virgin vs inseminated females, WT males lived longer when caged with refractory females than with virgin females. One possible explanation for this could be that WT males are able to recognise previously inseminated females, i.e. females that are refractory to insemination, either through behavioural traits or chemical cues, and do not expend energy courting such females, whereas the genetically modified males may not be able to identify refractory females before investing a substantial amount of energy. However, it is conceivable that in the field behavioural traits of females, which could be influenced by the confined mating arena provided in the laboratory, such as dispersal of mated females in search of breeding sites, may influence a male's ability to distinguish between receptive and refractory females.

The reduced insemination capacity and the higher cost of mating to OX513A males established in this paper is evidence of possible fitness deficits in this line. However, the males of the two lines inseminated a similar number of females over the first three days, following which the performance of the genetically modified line declined. Therefore, the time after release in which OX513A males are effective may be somewhat shorter, yet this in itself does not exclude their potential use in a control programme, as long as a strategy of frequent releases was adopted. Furthermore, this initial similarity in insemination capacity may suggest that males of both lines have comparable initial sperm (or energy) reserves, but that OX513A males are less able to regenerate capacity after exhaustion. Dissection and sperm quantification methods [31] could possibly be used to confirm this hypothesis. Moreover, in a sterile-male release programme, there will be a large excess of males relative to females, e.g 10∶1 ratio [26], [32]. Consequently, if females mate only once the average life-time number of successful copulations is likely to be low, perhaps 0.1. While there may be considerable variation around this mean, very few males may be affected by sperm depletion. One question that must still be considered with regard to their suitability for release though, is the relative competitive ability of OX513A males in direct competition with wild type males. Fitness deficits observed in the laboratory assays described above may be more pronounced when (i) in direct competition for females and (ii) in competition with field-bred mosquitoes. Further analysis of this genetically modified line's potential effectiveness through cage and field trials is desirable.
